# The London Life Table

**Published:** 1902-11-08

**Authors:** 


### The Hospital.

A Journal of

The Medical Sciences and Hospital Administration.

Vol. XXXIII.—No. 841.] Edited by Sir Henry Burdett, K.C.B., and Solomon C. Smith, M.D., M.R.C.P. [November 8, 1902.

### The London Life Table.

Dr. Shirley Murphy has compiled from the records of public health during the ten years between 1891–1900, a life table which shows the position which the metropolis holds as a place of residence in the health scale. Roughly speaking, London is not a bad place to live in, though the duration of life among Londoners is not quite as long as the average duration of life all through England and Wales. It takes a still lower place when compared with a specially healthy town, such as Brighton; but, on the other hand, it stands out favourably in comparison with other large towns, such as Glasgow and Manchester. "London" is used in Dr. Murphy's table to mean the Registration County of London, and therefore includes the healthy suburbs as well as the unhealthy slums. All classes are included in the estimate; therefore comparison with towns of lesser magnitude, where rich and poor do not live so far apart, is quite legitimate. As this is the first extended life table that has been drawn up for London—though there have been more compendious ones made for earlier decades—its value as a basis for future comparisons is even greater than its importance as indicating the present sanitary condition of the city. Certain allowances have, however, to be made before the figures given can be regarded as correct. In the first place, many invalids come to London for special medical or surgical treatment, of whom an appreciable number die, thus increasing the death-rate. Against this must be set the fact that London, like other large cities, draws to itself a great many healthy young men and women, whose power of resistance to the influence of disease helps to raise the life average. Even when some of these break down it is common for them—especially the young women, such as domestic servants, who have not made a permanent home in the Metropolis—to return home, and if they die, their death is not reckoned as an item in London's mortality. As in 1901, about 43 per cent. of the men and 45 per cent. of the women living in London were natives of other places, some allowance must be made for those of their number who go home to die. In addition, there are institutions of various kinds—convalescent homes, asylums, and workhouses, which are outside the registration area, but most of the inmates of which have been London residents. Deaths in these places also should be, but are not, reckoned in London's death-roll.

The inferiority of London as a place of residence compared with districts which are set down as specially healthy, affects chiefly, as might be expected, young children. In early manhood and womanhood, it would seem as if London could hold its own with the healthiest place, and come out better than the country as a whole. And as the general average of survival, especially among children, has risen in London during the last ten years, it is possible that it has done so elsewhere.

The increase in expectation of life is most marked among children. Until we are quite sure to what extent other places have participated in the improvement it is not well for London to plume itself too much. It is curious to note that the increased vitality that has developed during the last ten years is less marked in the case of women than in that of men, except in the first five years of life, when it is a shade greater.

The question of the greater vitality which girls are supposed to possess has some interesting lights thrown upon it by this life table. It is a commonplace of nurses that boys are more difficult to rear than girls. So far as the first year of life is concerned, this is true. But thereafter, until the age of 13, the deaths among girls exceed, though often only by a little, the deaths of boys. This shows that the popular notion that a girl is somehow of finer and more delicate make than a boy is something more than a sentimental fancy. But after that, up to the age of 64, the death-rate among men, age for age, is greater than that of women. Thus it is during the active period of life that men die more readily than women, presumably because they are more exposed to hardship and accident. Only in the first year of life do they seem to have any real innate physical superiority over their brothers. If this assumption be true it would be interesting to discover if such women as nurses, who expose themselves to risks as freely as most men, show a higher death-rate than women as a whole. This is only one of many questions which the table raises but does not answer One would fain have the mortality at different ages in different trades shown. Such tables have a distinct practical value, for when two or more quinquennial or decennial estimates have been made, they furnish a basis of comparison to show whether the efforts now being made to improve the sanitary conditions under which our workers live are really effecting their end. Meanwhile Londoners of to-day will feel satisfaction in the thought that their expectation of life is at least a little greater than was that of people of their present age ten years ago.

